# *Eurosurveillance* annual theme 2024: Editors inviting submissions on changing urban environments and effects on infectious diseases and their epidemiology, surveillance, prevention and control

**DOI:** 10.2807/1560-7917.ES.2023.28.37.2309145

**Published:** 2023-09-14

**Authors:** Eurosurveillance editorial team

**Affiliations:** 1 European Centre for Disease Prevention and Control (ECDC), Stockholm, Sweden

**Keywords:** urban environment, infectious disease

Cities are constantly evolving; also in Europe, organisational and infrastructural changes are being considered or initiated to reach sustainable development goals (SDGs) by 2030 [1] and there are attempts to improve resilience to expected or unexpected hazards [2]. These endeavours, and future steps to deal with environmental and socio-demographic issues, such as reducing the environmental footprint, climate action, addressing urban segregation/inequalities and changing population size, as well as keeping prepared for emergencies [2] may entail profound transformations of urban environments.

Transformations, to name just a few among many, might involve public transport optimisation, reducing environmental health inequalities [2,3], curbing greenhouse gas emissions (through home insulation [4]; cycle lanes/walking avenues), creating green spaces [1,5] and recreational areas near water [6,7], or increasing flood resilience (sewerage/storm water channels, or water traps) [8-12].

The ongoing and the SDG/resilience-related transitions in European cities require reflection on their potential consequence on infectious diseases epidemiology. How will these affect, for example, respiratory infections, vector-/waterborne diseases, or antimicrobial resistance? As these transitions may impact communicable disease surveillance prevention and control activities, it is important to establish in what way such activities will need to adapt or take advantage of the city transformation movement. For instance, for surveillance, will more layers of epidemiological data on infectious diseases need to be collected (e.g. integration of wastewater surveillance) and analysed in new ways?

For our 2024 annual theme, we invite article submissions relevant for Europe, that:

- address how urban environment changes toward SDGs and resilience are affecting or might affect infectious disease epidemiology, surveillance, prevention and control;
- address if or how communicable disease surveillance, prevention and control activities may have to adapt to these urban changes or to innovate to remain responsive or become more effective;
- include relevant examples of urban transitions toward SDGs and/or emergency preparedness, which have taken infectious disease epidemiology into account, or examples of adapted surveillance, prevention and control of communicable disease as a result of such urban transitions.

If you would like to submit a paper or ask for more information, please consult our instructions for authors regarding article types or contact the editorial team at  eurosurveillance@ecdc.europa.eu.
